# The Association between Depressive Symptoms and Non-Psychiatric Hospitalisation in Older Adults

**DOI:** 10.1371/journal.pone.0034821

**Published:** 2012-04-04

**Authors:** A. Matthew Prina, Dorly Deeg, Carol Brayne, Aartjan Beekman, Martijn Huisman

**Affiliations:** 1 Department of Public Health & Primary Care, Institute of Public Health, University of Cambridge, Cambridge, United Kingdom; 2 Department of Epidemiology & Biostatistics and the EMGO Institute for Health and Care Research, VU University Medical Center, Amsterdam, The Netherlands; 3 Department of Psychiatry, VU University Medical Center, Amsterdam, The Netherlands; 4 Department of Sociology, VU University, Amsterdam, The Netherlands; Federal University of Rio de Janeiro, Brazil

## Abstract

**Background:**

It is known that people who suffer from depression are more likely to have other physical illnesses, but the extent of the association between depression and non-psychiatric hospitalisation episodes has never been researched in great depth. We therefore aimed to investigate whether depressed middle-aged and older people were more likely to be hospitalised for causes other than mental illnesses, and whether the outcomes for this group of people were less favourable.

**Methods & Findings:**

Hospital events from 1995 to 2006 were obtained from the Dutch National Medical Register and linked to participants of the Longitudinal Aging Study Amsterdam (LASA). Linkage was accomplished in 97% of the LASA sample by matching gender, year of birth and postal code. Depression was measured at each wave point of the LASA study using the Centre for Epidemiologic Studies Depression (CES-D). Hospital outcomes including admission, length of stay, readmission and death while in hospital were recorded at 6, 12 and 24 months intervals after each LASA interview. Generalised Estimating Equation models were also used to investigate potential confounders. After 12 months, 14% of depressed people were hospitalised compared to 10% of non-depressed individuals. There was a 2-fold increase in deaths while in hospital amongst the depressed (0.8% vs 0.4%), who also had longer total length of stay (2.6 days vs 1.4 days). Chronic illnesses and functional limitations had major attenuating effects, but depression was found to be an independent risk factor for length of stay after full adjustment (OR = 1.33, 95% CI: 1.22–1.46 after 12 months).

**Conclusions:**

Depression in middle and old age is associated with non-psychiatric hospitalisation, longer length of stay and higher mortality in clinical settings. Targeting of this high-risk group could reduce the financial, medical and social burden related to hospital admission.

## Introduction

Although adults aged 65 and over encompass for only a small percentage of global populations, they account for over 45% percent of all hospital admissions in England [1]. A rise in the number of older adults will have dramatic consequences on the already strained and saturated national health services. For that reason, maintaining individuals in home settings, reducing preventable admissions, has been on the agendas of UK health policy makers for several years, yet population evidence concerning major contributors of hospitalisations is still not well understood.

One of the largest contributors to the worldwide burden of morbidity is depression [2], which might be related to a considerable part of hospital admissions in developed countries too. Depression is a common mental disorder that is widespread amongst the older population and that is associated with physical co-morbidities, lower quality of life and disability [3], [4], [5]. Although it is known that there is a strong association between depression and chronic conditions, it is not yet clear whether this indeed translates into higher numbers of hospital admissions and worse hospital outcomes, as no large epidemiological studies have been conducted to investigate this.

The absence of evidence can partially be blamed on lack of comprehensive medical registers that are linkable to other datasets. Medical registers on their own have in fact poor sensitivity for depression, as a large majority of depressed people living in the community are not formally diagnosed and their mental health statuses are not recorded. Therefore, an external population representative dataset, where mental health status is measured, is essential for this type of research. The existence of a national medical register in the Netherlands, where data linkage was possible, allowed us to carry out this research.

Several studies have investigated the relationship between depression and hospital outcomes in specific populations (e.g. cardiovascular patients, diabetes patients, etc.) [6], [7], [8], [9], [10] and most have reported positive associations, however depression was measured at admission in the majority of these. To our knowledge, this is the only study that measured depressive symptoms prospectively without focusing specifically on one cause of hospital admission, and following a large number of middle-aged and older participants for up to 24 months.

We aimed to assess whether depressed middle-aged and older adults were more likely to be hospitalised for causes other than mental illnesses. We also assessed differences in hospital outcomes, namely length of stay, hospital mortality and re-admissions between depressed and non-depressed individuals.

## Methods

### Sources of data

Participants were selected from the Longitudinal Aging Study Amsterdam (LASA) [11], a longitudinal study that includes a representative random sample of Dutch adults aged between 55 and 85 at baseline. The methods and characteristics of this sample have been described in great detail in previous publications [12]. Data collection in LASA started in 1992–1993 (LASA baseline) with follow-ups at roughly 3 years intervals. In 2003/2003, ten years after the baseline measurement of the first LASA cohort, a new cohort was started with adults aged between 55 and 65 years at that time. This new population sample was selected using the same procedures and sampling frame as the initial LASA cohort. Respondents of both the initial and the second LASA cohort have been followed-up together since then.

**Figure 1:**
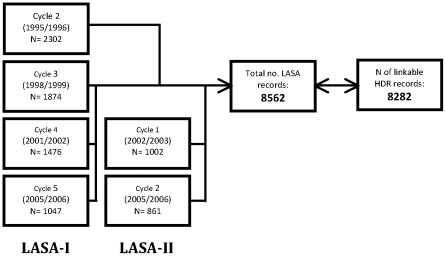
Number of linkable LASA records by wave and cohort.

To address our research question we included LASA participants who had at least one interview between 1995 and 2006, because access to complete medical records was only available for that time frame. A total of 8560 observations across different waves and cohorts were recorded, as illustrated in **figure 1**.

The Dutch Hospital Discharge Register (HDR) [13] is a register of hospital admissions. All general and academic hospitals and most single-specialty hospitals, with the exception of epilepsy clinics and long-stay centres for rehabilitation and asthma treatment, are included in this register. Thus, the register provides a nearly complete coverage of hospital inpatient treatments in the Netherlands. All clinical and day admissions are registered based on a uniform registration system. The data include admission and discharge dates, extensive treatment information (including about 10,000 medical procedures), and death.

Information from the HDR about individuals can be linked to other datasets via the Municipal Population Registration (MPR). The MPR contains basic information like date of birth, registered partners, and postal code for everyone listed in a Dutch municipality. Linkage of the HDR to the MPR occurred on the basis of a set of identifying variables: postal code, date of birth and gender [13]. The LASA dataset was also linked to the MPR, by Statistics Netherlands especially for our research, so that LASA data could be linked with information from the HDR as well. Linkage of LASA data with the MPR also occurred on the basis of identifying background variables. Participants from LASA were linked to data from the HDR at each wave and hospitalisation was followed up for 6, 12 and 24 months. Only records that had a full 24 months follow up, or censored data due to death, were included. Linkage was fully achieved for 8282 records, 97% of the initial sample. Consent forms for accessing medical records were signed by the LASA participants and the study was approved by the Medical Ethical Committee of the VU University Medical Centre.

### Measures and outcomes

Depression, physical co-morbidities, socio-demographics and functional limitations were recorded in LASA during each wave.

*Depressive symptoms* were measured at each wave using the Centre for Epidemiological Studies Depression Scale (CES-D), using a cut-off score of 16 points. This scale has been validated and been found to have good specificity and sensitivity for depression and to be a satisfactory instrument to measure depression severity in older people [14], [15], [16], with similar psychometric properties in its Dutch version [17]. Criterion validity for major depression was also shown to be very high, with a sensitivity of 100% and a specificity of 88% [18], and with very little overlap with symptoms of physical morbidity [19], [20].

Socio-demographics co-variates included *age*, *sex* and *level of education*. This was classified as no education, elementary/lower, intermediate or higher education. The total number of *chronic illnesses* ranged from 0 to 9 and was identified by asking questions on arthritis, peripheral atherosclerosis, cardiac disease, diabetes, malignant neoplasms, chronic lung disease, stroke, and a maximum of two other chronic diseases. These self-reported answers of LASA respondents were shown to correspond well with information from general practitioners [21]. A questionnaire on difficulty experienced with several activities (e.g. cutting own toenails, using public transport and walking up & down stairs) was used to measure *functional limitations*. These three questions were selected from the validated OECD Questionnaire [22], after showing good internal reliability (0.73) and being a good proxy for functional limitations [23]. Functional limitations were categorised into four severity groups according to the response to the three questions.

*Smoking* was categorised in current use, past use or no use. Alcohol consumption was measured with a questionnaire developed for the Netherlands Health Interview Survey [24] and was classified into two categories (excessive/severe vs not excessive/severe) according to the Garretsen Index of Present Alcohol Use [25]. Excessive alcohol use is defined as drinking at least once a week six glasses or more, or 21 days per month drinking four or more glasses.

The *outcomes* that were investigated were hospital admission (0 = non admitted, 1 = admitted into hospital), mean total length of stay across the different hospitalisation episodes, death while in hospital, number of readmissions (excluding no admissions) and type of admission. This latter was either day care admission (i.e. day hospital) or full overnight admission.

### Statistical analyses

After pooling the data from each wave and cohort, we tabulated hospital outcomes by depression status for three follow-up periods: 6, 12 and 24 months. Differences in the outcome variables by depression status were evaluated with Chi-Square X^2^ tests for categorical variables.

Due to the longitudinal nature of our observations, Generalised Estimating Equations (GEE), which take into account the dependency of the repeated measurements within persons, were used to investigate risk factors. Based on the nature and distribution of the outcome we decided what type of GEE regression function to use (e.g. logit vs negative binomial).

All the models were initially adjusted for age and sex, before fully adjusted models were run. The most conservative models are reported. In order to determine whether a potential confounder should be included in the final model, we added one factor at the time to see whether it showed a significant association with one or more of the outcomes.

Moreover, we selected all the participants (n = 2251) who had fully linkable hospital events from the first LASA cohort (wave 1995–1996) and plotted Kaplan-Meier survival curves (36 months) using hospital admission and death in hospital as outcomes. Data was only gathered from the first cohort to maintain a high consistency and to exclude that introduction of health policies or changes in health services could affect the results. Moreover a full follow up of 36 months was not present for all participants in the second cohort. Log rank tests were also performed to compare the cumulative admission and mortality rates between depressed and non-depressed individuals.

## Results

Depressed older adults were more likely to be older, be women, have lower socio-economic status, and have poorer physical health than individuals free of depressive symptoms (**table 1**). Two years after the interview, 27% of depressed older adults were admitted into hospital at least once, compared to only 23% of non-depressed participants (**table 2**). These differences were statistically significant also at 12 and 6 months, as confirmed by the X^2^ p values, which were lower than 0.0010. Depressed older adults were also twice more likely to die while in hospital than non-depressed individuals (p values<0.05).

**Table 1:** Population characteristics of linkable observations.

*Characteristics*	*No Depressive symptoms (frequency)*		*Depressive symptoms (frequency)*	
**Age group**				
54–65	2161	(30.99)	314	(24.01)
65–74	2287	(32.79)	336	(25.69)
74–85	1925	(27.60)	452	(34.56)
85+	601	(8.62)	206	(15.75)
**Sex**				
Male	3383	(48.51)	391	(29.89)
Female	3591	(51.49)	917	(70.11)
**Education**				
No	2189	(31.39)	609	(46.67)
Elementary/Lower	3486	(49.99)	539	(41.30)
Intermediate	962	(13.80)	125	(9.58)
Higher	336	(4.82)	32	(2.45)
**Smoking**				
Never	1786	(31.71)	400	(36.53)
Used to	2820	(50.07)	453	(41.37)
Smokes	1026	(18.22)	242	(22.10)
**Alcohol** (potential alcohol problem)				
No	5366	(95.28)	1052	(96.07)
Yes	266	(4.72)	43	(3.93)
**Functional Limitations**				
No	3755	(54.79)	288	(22.41)
2 without	1572	(22.82)	249	(19.38)
1 without	947	(13.74)	321	(24.98)
All without	596	(8.65)	427	(33.23)
**Chronic illnesses**				
Mean number	1.67	(1.64–1.70)	2.50	(2.41–2.58)
**Anxiety**				
No	5831	(95.14)	555	(47.76)
Yes	298	(4.86)	607	(52.54)

**Table 2:** Hospital admissions and other hospital outcomes for depressed (CES-D>16) and non-depressed (CES-D<16) participants.

*Hospital outcomes*	*24 Months*				*12 Months*				*6 Months*			
	*Not Depressed*		*Depressed*		*Not Depressed*		*Depressed*		*Not Depressed*		*Depressed*	
**Hospital Admission**												
No (frequency)	5371	(77.01)	952	(72.78)	6252	(89.65)	1131	(86.47)	6461	(92.64)	1171	(89.53)
Yes (frequency)	1603	(22.99)	356	(27.22)	722	(10.35)	177	(13.53)	513	(7.36)	137	(10.47)
**Death in hospital**												
No (frequency)	6866	(98.45)	1270	(97.09)	6946	(99.6)	1297	(99.16)	6957	(99.76)	1299	(99.31)
Yes (frequency)	108	(1.55)	38	(2.91)	28	(0.40)	11	(0.84)	17	(0.24)	9	(0.69)
**Day Care**												
No (frequency)	1179	(73.55)	278	(78.09)	507	(70.22)	137	(77.40)	346	(67.45)	105	(76.64)
Yes (frequency)	424	(26.45)	78	(21.91)	215	(29.78)	40	(22.60)	167	(32.55)	32	(23.36)
**Number of readmissions**												
Mean (95% CI)	2.10	(1.99–2.20)	2.25	(2.02–2.48)	1.96	(1.84–2.08)	1.99	(1.77–2.22)	1.86	(1.74–1.98)	1.90	(1.66–2.13)
**Total length of stay**												
Mean (95% CI)	3.51	(3.21–3.81)	5.31	(4.37–6.26)	1.40	(1.21–1.59)	2.62	(1.98–3.25)	0.90	(0.76–1.05)	1.73	(1.23–2.23)

There were not any major differences in the number of re-admissions at any of the follow-up times. However, there were marked variations in mean total length of stay between the two groups. At 24 months, hospitalised depressed individuals spent on average 5.3 (95% CI: 4.4–6.3) days in hospital compared to only 3.5 days (95% CI: 3.2–3.8) for participants without depression. Similar differences were found at 6 and 12 months (**table 2**). The last outcome that was investigated was related to the type of admission. Two types of admission were recorded: day hospital or full admission. Differences in the types of admissions were found at 6 months (p value<0.05) but not at 12 (p = 0.07) or 24 months (p = 0.09). Although during the latter two follow-up periods the variations were only marginally significant depressed older adults had a lower ratio of day hospital to full admissions compared to their depressed peers.

### Confounders and associated risk factors

Generalised Estimating Equations [26], [27] were used to investigate potential risk factors involved in explaining the association between depression and hospital outcomes (**table 3**). Unadjusted Odds Ratios (ORs) for the depressed group were highest for the mortality outcome (6 months ORs = 2.8, 95% CI: 1.26–6.37), but were also above one for all the other outcomes. After adjustment for age and sex, depression was still found to be an independent risk factor for hospital admission, longer length of stay and death. After full adjustment for socio-economic status and physical co-morbid conditions, the effect of depression was attenuated. However, the ORs remained high for the mortality outcome, and depression was still found to be an independent risk factor for total length of stay (6 months ORs = 1.17, 95% CI: 1.07–12.9; 12 months ORs = 1.33, 95% CI: 1.22–1.46). Similar outcomes were found at 24 months follow up (**data not shown**). Separate regressions were also fitted to assess what factor led to the largest attenuation of the associations. Adjusting for the number of functional limitations consistently had the biggest impact on the Odds Ratios (**data not shown**).

**Table 3:** GEE (Generalised Estimating Equation) models for the independent effect of depression (CES-D>16) on different. outcomes.

*Outcomes*	*Hospital Admission (function type: logistic)*				*Total Length of Stay (function type: negative binomial)*				*Dying During Hospital Stay (function type: negative binomial)*			
*ORs (95% CI)*	*12 months*		*6 months*		*12 months*		*6 months*		*12 months*		*6 months*	
**Model 1**												
Depression	1.35	(1.13–1.62)	1.47	(1.20–1.80)	1.86	(1.73–2.00)	1.88	(1.74–2.03)	2.10	(1.05–4.24)	2.84	(1.26–6.37)
**Model 2**												
Depression	1.34	(1.11–1.60)	1.47	(1.20–1.81)	1.79	(1.67–1.93)	1.78	(1.65–1.93)	1.96	(0.96–4.02)	2.67	(1.16–6.15)
Age	1.02	(1.01–1.03)	1.02	(1.01–1.03)	1.04	(1.04–1.04)	1.04	(1.04–1.04)	1.05	(1.02–1.09)	1.08	(1.03–1.13)
Sex	0.78	(0.67–0.90)	0.77	(0.65–0.91)	0.87	(0.82–0.92)	0.87	(0.81–0.92)	0.62	(0.33–1.18)	0.42	(0.18–0.94)
**Model 3**												
Depression	1.01	(0.83–1.22)	1.07	(0.86–1.33)	1.33	(1.22–1.46)	1.17	(1.07–1.29)	1.35	(0.58–3.17)	1.90	(0.68–5.28)
Age	1.00	(1.00–1.01)	1.00	(0.99–1.01)	1.04	(1.03–1.04)	1.04	(1.04–1.05)	1.20	(0.97–1.06)	1.04	(0.98–1.11)
Sex	0.70	(0.60–0.82)	0.69	(0.58–0.82)	0.80	(0.74–0.86)	0.80	(0.74–0.87)	0.70	(0.32–1.53)	0.58	(0.21–1.56)
Education	1.00	(0.91–1.10)	0.98	(0.88–1.10)	0.93	(0.89–0.98)	0.94	(0.89–0.98)	0.87	(0.53–1.44)	0.80	(0.42–1.53)
Chronic illnesses	1.20	(1.14–1.27)	1.23	(1.16–1.31)	1.16	(1.13–1.20)	1.23	(1.19–1.26)	1.21	(0.93–1.57)	1.18	(0.85–1.65)
Functional limitations	1.25	(1.15–1.35)	1.26	(1.14–1.38)	1.29	(1.24–1.33)	1.30	(1.24–1.35)	1.46	(0.98–2.18)	1.42	(0.86–2.38)
Smoking	n/a		n/a		1.20	(1.14–1.26)	1.20	(1.14–1.28)	1.93	(1.12–3.32)	2.13	(1.07–4.22)
Alcohol problem	n/a		n/a		1.29	(1.10–1.52)	1.43	(1.20–1.70)	0.70	(0.92–5.41)	1.28	(0.16–10.36)

**Figure 2:**
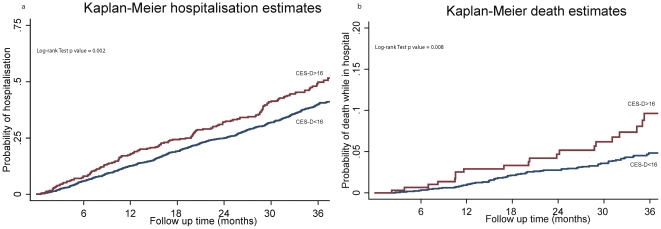
Keplan-Meier survival curves for hospitalisation and death while in hospital. Red line: depressive symptoms (CES-D>16). Blue line: no depressive symptoms (CES-D<16).

Hospital admissions and deaths while in hospital were significantly different between groups on Kaplan-Meier analyses (**figure 2**). The p values of log-rank tests for the hospital admission and death were respectively equal to 0.002 and 0.008.

## Discussion

This is the first study of this scale that clearly identifies an association between depressive symptoms and non-psychiatric hospitalisation episodes and that does not specifically focus on specific hospitals, wards or diseases.

Our study confirmed that depression is associated with hospital admission and with worse hospital outcomes in a middle-aged and older depressed Dutch population. By introducing covariates in our models, the Odds Ratios were reduced between 45–97%. The association between depression and admission was mostly explained by higher physical co-morbidities and functional limitations but depression remained an independent risk factor for longer length of stay, and had a strong association with mortality as well. Several explanations could be put forward to explain the association between depression and poorer hospital outcomes.

It is known that depressed individuals often do not follow medical recommendations for underlying medical illnesses and have lower treatment compliance [28], [29], [30]. Although this is likely not to be of major concern during hospitalisation, it could affect those individuals while in the community. Those persons may consequently present to a hospital with a later or more severe stage of disease that requires more invasive treatment and a higher chance of adverse outcome that would affect length of stay and mortality. Unfortunately we could not tease out severity or type of treatment received during hospitalisation, but the fact that more depressed participants were admitted for full overnight admissions rather than for day care seems to suggest that acuteness and severity might be higher in this group. It is also possible that longer LOS may be the result of the discovery of the mental disorder during admission, requiring the involvement of liaison psychiatry services that could lengthen the stay, while trying to optimise the psychiatric illness.

The most apparent explanation for this association lies in the strength of association between depression and chronic conditions. Several mediators (e.g. limbic-hypothalamic-pituitary-adrenal axis alterations, pro-inflammatory milieu, decline in glucocorticoid receptor function, etc.) occurring in depressed individuals that increase the likelihood of physical morbidity have been described by Wolkowitz OM, et al. [31]. However, our findings suggest that the relationship between depression and hospitalisation is not solely mediated by chronic conditions and might result from the synergic action of several different pathways. Our findings also suggest that functional limitations may be on the pathway to hospital outcomes in depressed patients, or conversely, that functional limitations are causing depressive symptoms that in turn lead to poor hospital outcomes. The causal association between depression and disability has been shown to be bidirectional [32], [33].

### Strengths

The major strengths of our study stand in the large cohort, in its longitudinal nature, and in the excellent validity of the linked registry. The latter was demonstrated in a random sample of records, showing that 84% of the main diagnoses (validated through medical record review by medical specialists) and 99% of the personal, admission and discharge data were correctly recorded in the hospital discharge register [34]. The longitudinal nature of the study allowed us to identify depression first, and assess hospital outcomes thereafter, thus avoiding ambiguities in causal direction. No studies have been able to investigate the association between depression and hospital outcomes in such detail and with such complete coverage.

### Limitations

This is a study conducted in a nationally representative sample of the Dutch middle-aged and older population. We believe that our findings could be easily applicable to other western developed countries. Even though there are differences in the prevalence of lifetime depression and impairment related to depression between western developed countries [35], the burden of morbidity due to depression is immense in all of them. However specifics about access to health care, treatment, and recognition of depression in older adults are likely to differ [36], which may affect the likelihood of depressed older adults to become hospitalised.

Depressive symptoms were only measured at the beginning of each wave. Considering the episodic nature of this disorder and the length of our follow up (up to 24 months), we are therefore not able to exclude classification bias (e.g. some participants free from the condition could have developed depression before hospitalisation, whereas remission could have occurred amongst some depressed participants).

The CES-D is a symptom rating scale, and although this measures clinically relevant depressive symptoms, it is not comparable to the definition used in the DSM classification [37]. Nevertheless, it has been demonstrated that the consequences of depressive symptoms in older adults are very similar to those of major depressive illnesses [38].

Finally, using a general population sample, compared to a sample with specific medical conditions, has its advantages but we should also emphasise its limitations. In a more homogenous sample a comparison of those depressed and not depressed is less likely to be confounded by differences in co-occurring medical conditions. Moreover, in this highly selected groups, measuring and controlling for the severity of the medical illness is easier to make compared to a general cohort.

### Implications & Conclusions

Although depression is so prevalent in middle-aged and older adults and has adverse effects on hospital outcomes, it is not often noticed and treated in general hospitals. Clinicians should be aware of the high prevalence of depression in older adults, and also of the importance of treatment options and early identification of depressive symptoms that could reduce hospitalisation, length of stay and improve survival.

It is known that the bulk of hospitalisations occur among older adults and that hospital care is the most costly form of care. Our findings strongly suggest that there are opportunities to drastically reduce costs related to admissions if case finding and treatment of depressive symptoms in older adults could be improved. Further evidence to support the previous claim is certainly needed as although it is known that screening paired with an organised system of depression care can improve depression outcomes [39], it is not clear whether physical outcomes could also be improved. Future research that includes a full economic analysis of this prevention approach is needed, together with studies aimed at investigating the role of clinical depression but also of anxiety, so often co-morbid with depression. A trial for case finding and effective treatment of depression in a high-risk group of older adults (e.g. comorbid depression and chronic medical illnesses), to see whether hospital outcomes could be improved, is also warranted. Although we solely focused on one direction of this association, this relationship is likely to be bi-directional, where those sick enough to be hospitalised are more often depressed, potentially creating a feedback loop between mental status and admissions into hospital. The investigation of this phenomenon deserves more attention. In conclusions, this study suggests that there is an association between depression, hospital admission and worse hospital outcomes, identifying a potential target to reduce preventable admissions and ensuing associated costs.
